# Lyme Borreliosis and *Borrelia spielmanii*

**DOI:** 10.3201/eid1207.060077

**Published:** 2006-07

**Authors:** Vera Maraspin, Eva Ruzic-Sabljic, Franc Strle

**Affiliations:** * University Medical Centre Ljubljana, Ljubljana, Slovenia;; † University of Ljubljana, Ljubljana, Slovenia

**Keywords:** Borrelia spielmanii, Lyme borreliosis, erythema migrans, case report, letter

**To the Editor:** A report on erythema migrans (EM) caused by *Borrelia spielmanii* in a recent issue of Emerging Infectious Diseases (*1*) was a stimulus for a review of data on this *Borrelia* species in patients with early Lyme borreliosis (LB). We report a patient with EM, examined at our LB outpatient clinic, from whom *B*. *spielmanii* was isolated from the skin lesion. The presence of this species was ascertained by using a 5S–23S spacer amplicon after digestion with *Mse*I and demonstration of fragments having sizes typical for *B*. *spielmanii* (106, 68, and 51 bp) (*2*).

A 69-year-old woman was examined on October 30, 1996, for a skin lesion on her left thigh. Her medical history indicated arterial hypertension, intermittent pain in the cervical and lumbar region due to spondylosis, frequent headaches and myalgias, and treatment of typical EM skin lesions at our LB outpatient clinic in 1992 and 1994; the latter lesions were culture positive for *Borrelia*. Fourteen days before examination, she noticed a small area of redness, accompanied by mild local itching, burning, and pain on her left knee. On examination, a 24 × 20–cm ringlike lesion was found on her left thigh. Basic blood tests did not show abnormal results, and a serum sample was negative for borrelial antibodies (immunofluorescence test using a *B*. *afzelii* skin isolate as antigen) (*3*). However, *B*. *spielmanii* was isolated from an EM skin biopsy specimen. The patient was treated with amoxicillin, 500 mg 3 times a day for 15 days. The skin lesion disappeared within 3 weeks, and a culture of a repeat skin biopsy specimen was negative for *Borrelia* 2 months after the first biopsy. Her clinical course during a 1-year follow-up was uneventful.

*B*. *spielmanii* was detected in the patient by a general approach we have used for several years. In all consenting patients, a skin specimen from an EM lesion is cultured for borreliae in modified Kelly medium before and, in case of a positive result, ≈2 months after antimicrobial drug treatment is started. Isolated strains are typed by using the 5S–23S spacer amplicon.

The findings in this report are generally consistent with those in other reports of adult patients with EM (*4**–**8*). One difference was that the patient did not report a tick bite at the site of the EM. Approximately two thirds of our patients with EM recalled a tick bite and ≈10% of patients treated for early LB had previously had EM (*4**–**8*).

Previous reports indicate several differences in patients with EM caused by *B*. *burgdorferi* and *B*. *afzelii* (*7*) and patients with EM caused by *B*. *afzelii* and *B*. *garinii* (*8**,**9*). Some of the findings in our patient are unusual and rarely found in those with early LB. However, the small number of patients infected with *B*. *spielmanii* (1 reported herein and 4 previously reported) does not allow any reliable conclusion to be made on differences in clinical manifestations of LB caused by *B*. *spielmanii* compared with those of other species.

Our results corroborate previous findings that *B*. *spielmanii* is a cause of LB in Europe. Thus, in addition to the Netherlands (*2*), Germany (*10*), and Hungary (*1*), LB caused by *B*. *spielmanii* is also present in Slovenia.
